# Atherosclerosis and Insulin Resistance: Is There a Link Between Them?

**DOI:** 10.3390/biomedicines13061291

**Published:** 2025-05-23

**Authors:** Alina Diduța Brie, Ruxandra Maria Christodorescu, Roxana Popescu, Ovidiu Adam, Alexandru Tîrziu, Daniel Miron Brie

**Affiliations:** 1 Department of Cell and Molecular Biology, “Victor Babes” University of Medicine and Pharmacy, Tudor Vladimirescu Street, No. 14, 300174 Timisoara, Romania; alina.brie@umft.ro (A.D.B.); popescu.roxana@umft.ro (R.P.); 2 ANAPATMOL Research Center, “Victor Babes” University of Medicine and Pharmacy, Tudor Vladimirescu Street, No. 14, 300174 Timisoara, Romania; 3 “Louis Țurcanu” Emergency Children Hospital, Doctor Iosif Nemoianu Street, No. 2, 300011 Timisoara, Romania; 4 Department of Medical Semiology, “Victor Babes” University of Medicine and Pharmacy, Eftimie Murgu Square, No. 2, 300041 Timisoara, Romania; 5 Department of Pediatric Surgery and Orthopedics, “Victor Babes” University of Medicine and Pharmacy, Eftimie Murgu Square, No. 2, 300041 Timisoara, Romania; 6 Cardiovascular Disease Institute Timisoara, Gheorghe Adam Street, No. 13A, 300310 Timisoara, Romania; alexandru.tirziu@umft.ro (A.T.); brie_daniel@yahoo.com (D.M.B.); 7 Department of Functional Sciences, “Victor Babes” University of Medicine and Pharmacy, Tudor Vladimirescu Street, No. 14, 300174 Timisoara, Romania; 8 Research Center of the Institute of Cardiovascular Diseases, Cardiovascular Disease Institute Timisoara, Gheorghe Adam Street, No. 13A, 300310 Timisoara, Romania

**Keywords:** insulin resistance, subclinical atherosclerosis, endothelial dysfunction, cardiovascular risk

## Abstract

Cardiovascular disease remains the leading cause of morbidity and mortality worldwide, especially in regions like Eastern Europe, South Asia, and Latin America. A significant portion of these cases (80%) is linked to atherosclerosis, which can lead to severe conditions like ischemic heart disease and stroke, with atherosclerosis (ATS) responsible for the majority of cases. This review explores the multifaceted relationship between insulin resistance (IR) and ATS, highlighting their roles as both independent and interrelated contributors to cardiovascular risk. ATS is characterized by lipid accumulation and chronic inflammation within arterial walls, driven by factors such as hypertension, dyslipidemia, and genetic predisposition, with endothelial dysfunction as a key early event. The early detection of subclinical ATS is critical and can be achieved through a combination of non-invasive imaging techniques—such as coronary artery calcium scoring and carotid ultrasound—and comprehensive risk profiling. IR, marked by impaired glucose uptake in liver, muscle, and adipose tissue, often precedes early diabetes and is associated with metabolic disturbances, including dyslipidemia and chronic inflammation. The diagnosis of IR relies on surrogate indices such as HOMA-IR, the QUICKI, and the TyG index, which facilitate screening in clinical practice. Compelling evidence indicates that IR independently predicts the progression of atherosclerotic plaques, even in non-diabetic individuals, and operates through both traditional risk factors and direct vascular effects. Understanding and targeting the IR–ATS axis is essential for the effective prevention and management of cardiovascular disease.

## 1. Introduction

Cardiovascular disease remains the predominant cause of morbidity and mortality globally, with a particularly high prevalence in regions such as Eastern Europe, South Asia, and Latin America [1,2]. A significant proportion of these cases (80%) are attributed to atherosclerosis (ATS), which can progress to ischemic heart disease, stroke, or peripheral arterial disease [3]. In developed nations, the incidence of atherosclerotic cardiovascular disease is on the decline, primarily due to the enhanced management of risk factors, including smoking, hypertension, and dyslipidemia and the implementation of primary prevention programs [4,5].

Insulin facilitates the uptake of glucose into cells, where it serves as an energy source. However, in certain conditions, cells may develop resistance to insulin, necessitating increased insulin secretion by the pancreas. Numerous studies have established a correlation between insulin resistance and ATS [6,7,8,9,10].

**Figure 1:**
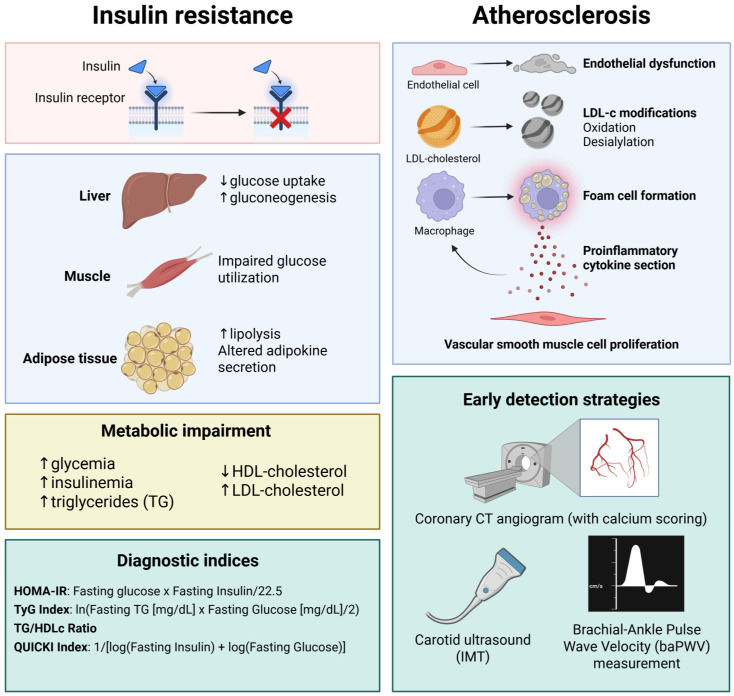
The connection between insulin resistance and atherosclerosis development and progression. Insulin resistance triggers gluconeogenesis since glucose uptake mediated by GLUT-3 is impaired. At the muscle level, due to impaired GLUT-2/4-mediated glucose uptake, glucose utilization is impaired. At the adipose tissue level, lipolysis is increased and adipokine secretion is altered, leading to a metabolic impairment characterized by hyperglycemia, initial hyperinsulinemia, increased LDL-cholesterol and trigyliceride levels, and reduced serum high-density lipoprotein (HDL)-cholesterol. In clinical practice, the TyG index is widely used as a screening method for insulin resistance. Alongside the TyG index, other indices such as the homeostasis model assessment of insulin resistace (HOMA-IR), the triglyceride/HDL-cholesterol ratio (TG/HDLc), and the quantitative insulin sensitivity check index (QUICKI) have also proved their utility in insulin resistance screening. Increased low-density lipoprotein (LDL)-cholestrol levels, in the presence of cardiovascular risk factors, leads to endothelial dysfunction and subsequent modifications of LDL-cholesterol particles, such as oxidation and desialylation, triggering a macrophage-mediated immune response. The most widely used paraclinical test for diagnosing subclinical atherosclerosis is coronary computed tomography (CT) angiogram (with calcium scoring), also suggested by the actual guidelines for improved risk stratification in coronary artery disease (CAD). Created in BioRender. Tirziu, A. (2025) https://BioRender.com/2wkbiot (accessed on 20 May 2025).

The following questions arise: How can subclinical ATS be diagnosed? What are the diagnostic criteria for insulin resistance? Is there a definitive link between insulin resistance and ATS? What interventions can be implemented to ameliorate insulin resistance? Is it possible to treat insulin resistance effectively? Could routine screening for insulin resistance become standard practice? Would the early detection of insulin resistance play a crucial role in the primary prevention of cardiovascular disease? (Figure 1)

In the following sections, we will review existing data and endeavor to address these questions to the furthest extent possible.

## 2. Atherosclerosis: General Considerations

Atherosclerosis is characterized by the accumulation of lipids within the arterial wall. It is a chronic inflammatory condition with focal manifestations that commences in childhood and progresses through a prolonged asymptomatic phase [11]. Recognizing that an intact endothelium is the most significant factor in preventing atherosclerosis is crucial. Various “keys” to disrupt the endothelial barrier represent risk factors, including hypertension, insulin resistance, diabetes mellitus, dyslipidemia, smoking, genetic predispositions, and pollution, which compromise the endothelial layer and facilitate intravascular lipid accumulation in the intima. However, the precise mechanism by which this accumulation triggers inflammation remains unclear [11,12]. In the initial stages of the atherosclerotic process, following the formation of lipid striations due to the infiltration of low-density lipoprotein (LDL) cholesterol particles into the arterial wall, a potentially reversible stage occurs with the formation of foam cells (macrophages that phagocytize LDL particles) and the initiation of the inflammatory response [13].

Recent research indicates that not all low-density lipoprotein (LDL) particles exhibit atherogenic properties. Specifically, the desialylation of LDL-cholesterol (LDLc) particles, has been shown to enhance their atherogenic potential [14,15]. These desialylated LDLc particles are smaller, denser, more electronegative, and exhibit an increased susceptibility to oxidation [15,16,17]. Similarly, the desialylation of high-density lipoprotein cholesterol (HDLc) molecules impairs their ability to extract lipids from peripheral tissues and transfer to the liver [18]. Furthermore, the role of inflammation in the ATS process has been increasingly recognized, providing a partial explanation for the occurrence of 50% of initial cardiovascular events in patients with normal LDLc levels [19,20,21]. Recent studies have proposed several mechanisms involved in the ATS process, highlighting the significance of genetic interactions, such as polygenic risk scores for coronary artery disease (CAD), which correlate well with plaque burden and can be utilized to assess cardiovascular risk in young patients [22,23,24,25]. These genetic factors interact with exogenous risk factors in the organs and the arterial wall [26]. However, the influence of hypertension, diabetes mellitus, and chronic kidney disease on ATS progression remains unclear, as does the beneficial role of exercise and the factors that confer protection in women [11].

## 3. Early Detection of Asymptomatic Atherosclerosis

Atherosclerotic lesions develop progressively and are frequently irreversible; however, some research suggests that lipid-rich soft plaques may be reversible. Consequently, early diagnosis and prevention are essential for effective ATS management. In alignment with this perspective, an article in the Journal of the American College of Cardiology (JACC) [27] advocated for a paradigm shift from addressing the late manifestations of ATS to prioritizing early detection and prevention, as identifying the disease in its subclinical stages may offer curative potential in some instances [28]. Insulin resistance generally manifests a decade or more before the clinical diagnosis of type 2 diabetes, and this period of hyperinsulinemia may contribute to atherogenesis by fostering the formation of fatty streaks and early atheromatous lesions. The early detection of ATS should encompass a comprehensive evaluation of risk factors, including the early identification of insulin resistance before the onset of diabetes mellitus, paraclinical analyses [complete lipid profile: low-density lipoprotein cholesterol (LDLc), high-density lipoprotein cholesterol (HDLc), triglycerides (TG), lipoprotein a (Lp(a)), high-sensitive C-reactive protein (hsCRP), glycated hemoglobin (HbA1c), creatinine clearance (Cl_creatinine_), von Willebrand factor (vWF), metalloproteinases, particularly the matrix metalloproteinase type 9 (MMP-9), and various cytokines], and non-invasive imaging techniques [29,30,31] such as coronary artery calcium scoring, carotid artery ultrasound with IMT measurement, femoral artery ultrasound, and ankle–brachial index measurement.

The calcium score, as determined by computed tomography (CT) using the Agatston method, is a diagnostic tool for identifying calcifications in the coronary arteries. Typically, calcifications emerge following the prolonged progression of the ATS process within the coronary arteries, which can be detected via CT imaging. Numerous studies have demonstrated a positive correlation between the calcium score, coronary artery stenosis, and ATS [32,33,34]. Caution is advised when interpreting scores in young patients or women, as an Agatston score of 0 does not entirely exclude the presence of atherosclerosis. Nonetheless, the likelihood of myocardial infarction or mortality remains low (less than 1%) in individuals with a calcium score of 0 [35]. This scoring method has been effectively integrated into the 10-year cardiovascular risk assessment model. The Heinz Nixdorf Recall Study and the Dallas Heart Study validated the MESA risk score [36,37,38]. Carotid IMT is a widely utilized marker for detecting subclinical ATS [39,40]. The absence of atherosclerosis in the carotid arteries does not preclude its presence in the coronary arteries, as evidenced by a recent meta-analysis that found no positive correlation between IMT and cardiovascular risk in the general population [41]. Some researchers have proposed that the carotid plaque score (CPs), rather than IMT, may better correlate with the risk of future events in patients with existing cardiovascular disease [42]. Another meta-analysis suggested that combined intima-media thickness (cIMT), which includes measurements from multiple carotid segments, correlates more effectively with future cardiovascular events than measurements limited to a single carotid segment [43].

Arterial stiffness can be assessed using brachial–ankle pulse wave velocity (baPWV) [44]. Some studies have indicated that baPWV does not possess a strong capacity to detect subclinical ATS [45,46], although its efficacy is enhanced when combined with other tests [47].

The integration of the coronary artery calcium score, the carotid plaque score (CPs), and brachial–ankle pulse wave velocity (baPWV) proves more effective in detecting subclinical ATS and predicting future cardiovascular events [48].

## 4. Insulin Resistance

Insulin resistance is defined by the impaired ability of insulin to regulate glucose metabolism in specific tissues, notably the liver, skeletal muscle, and adipose tissue, which fail to absorb glucose from the bloodstream, resulting in hyperglycemia. Insulin resistance can be either acquired, due to factors such as the accumulation of perivisceral and subcutaneous adipose tissue, physical inactivity with advancing age, and the use of certain medications, or congenital, as seen in conditions like polycystic ovary syndrome, Alstom syndrome, Rabson–Mendenhall syndrome, and Werner syndrome [49,50]. A sex difference is observed in insulin resistance, with a lower incidence in premenopausal women compared to men [51]. The primary consequences of insulin resistance include the inhibition of lipolysis in adipose tissue, reduced glucose utilization by muscles, and the inhibition of gluconeogenesis in the liver, leading to disturbances in lipid metabolism, such as increased triglycerides, decreased HDLc with modest disturbances in LDLc [52], impaired glucose tolerance, and hyperinsulinism [53]. Insulin resistance occurs significantly before it manifests as prediabetes or type 2 diabetes mellitus [54], and the patient remains asymptomatic for an extended period of time.

Insulin resistance is typically diagnosed using the hyperinsulinemic–euglycemic glucose clamp technique, as outlined by DeFronzo [55]. While precise, this method is labor-intensive and primarily utilized in specific clinical trials, posing challenges for routine clinical practice [56,57]. Consequently, in clinical settings, surrogate indices are generally employed (Table 1). Among these, the homeostatic model assessment for insulin resistance (HOMA-IR) is frequently used. This involves determining fasting blood glucose and insulin values, with HOMA-IR calculated as fasting blood glucose (mmol/L) × fasting insulin (mIU/L)/22.5. A HOMA-IR value exceeding 2–2.5 typically indicates insulin resistance, although this threshold may vary based on the population and clinical context. For instance, a study examining the prevalence of insulin resistance in a French population utilized a HOMA-IR cut-off value of 3.8 [58]. Conversely, the Bruneck study, which investigated insulin resistance prevalence in individuals with metabolic disorders, employed a HOMA-IR cut-off value of 2.77 [59].

(1) $$HOMA-IR=\frac{Fasting\;glucose\left(mg/dL\right)}{Fasting\;insulin\left(pmol/L\right)}\times 22.5$$

The triglyceride-to-high-density lipoprotein cholesterol (HDLc) ratio (TG/HDLc ratio), with a threshold greater than 3.5 in men and 2.5 in women, is a strong indicator for diagnosing insulin resistance [60,61]. However, some researchers have noted its applicability primarily in diagnosing insulin resistance among overweight Caucasians but not in African Americans [60,62]. An alternative index utilized in clinical practice is the QUICKI, calculated as 1/[log fasting insulin (μU/mL) + log fasting glucose (mg/dL)], which provides a more accurate characterization of insulin resistance compared to the HOMA index [63,64].

(2) $$QUICKI=\frac{1}{[\log\left(fasting\;insulin\left(\frac{\mu U}{mL}\right)\right)+\log\left(fasting\;glucose\left(\frac{mg}{dL}\right)\right]}$$

Another index, the Matsuda index, incorporates insulin and fasting glucose levels along with glucose tolerance test results in its calculation formula (10,000/√(fasting G × fasting I) (mean G × mean I)), serving as an indicator of insulin sensitivity [65]. A study conducted in a non-diabetic Finnish population demonstrated that the Matsuda index exhibited superior sensitivity to the HOMA index for diagnosing insulin resistance in this cohort [66].

(3) $$Matsuda\;index=\frac{10,000}{\sqrt{fasting\;glucose\left(\frac{mg}{dL}\right)\times fasting\;insulin\;\left(\frac{\mu U}{mL}\right)\times mean\;glucose\left(\frac{mg}{dL}\right)\times mean\;insulin\;(\frac{\mu U}{mL})}}$$

Recently, a novel marker, the triglyceride glucose index (TyG), was proposed, calculated as TyG index = ln (fasting triglycerides [mg/dL] × fasting glucose [mg/dL]/2) [67,68]. The development of this index was motivated by the high cost and impracticality of large-scale insulinemia determination. The TyG index was introduced as an alternative for diagnosing insulin resistance, with various cut-off values proposed: >ln 4.68 (sensitivity of 96.5%, specificity of 85%) [69]; >ln 4.66 (sensitivity of 86.2%, specificity of 44.1%) [70]; >ln 4.49 (specificity of 82%, sensitivity of 82.6%) [71]. This index has also shown a strong correlation with 10-year cardiovascular risk [72,73], atrial fibrillation risk, particularly in women [74,75], coronary calcium score [76], subclinical atherosclerosis and arterial stiffness [77], the prevalence and severity of coronary heart disease [78,79], metabolic syndrome in obese Caucasian patients [80,81], metabolic syndrome in Asian patients [82], the general population with or without metabolic syndrome [81], and non-alcoholic fatty liver disease [83,84,85]. Consequently, the TyG index is a marker for screening and diagnosing various pathologies primarily associated with insulin resistance and metabolic syndrome [86]. Some studies have indicated that the TyG index predicts the development of atherosclerosis more effectively than HOMA-IR, as evidenced by the intima-media thickness (IMT) index and coronary artery calcium score [87,88]. It is important to note that the HOMA-IR index is not applicable to patients with diabetes mellitus who are undergoing insulin treatment. The TyG index is employed in these cases [89].

The lipid accumulation product (LAP) index has been proposed as an alternative measure for assessing insulin resistance. This index was developed to describe the accumulation of subcutaneous adipose tissue more accurately and is considered to provide a more precise assessment of obesity risk compared to the body mass index (BMI). The LAP index is calculated using waist circumference and fasting triglyceride levels, with the formula LAP = (WC-65) × TG for men and (WC-58) × TG for women [90]. Subsequent studies have demonstrated that the LAP index offers superior estimates of 10-year cardiovascular risk compared to BMI, waist circumference, the waist-to-hip ratio, and the waist-to-height ratio [91]. Although the LAP index is not traditionally used as a measure of insulin resistance, it may offer a more effective assessment of insulin resistance risk than HOMA-IR or the QUICKI, as evidenced by a study conducted on a non-obese (BMI < 21 kg/m^2^) normoglycemic male population in Asia [92]. Furthermore, recent research has indicated that the LAP index serves as a reliable indicator for diagnosing metabolic syndrome in the obese adult population, outperforming the body adiposity index (BAI) [93], which is calculated as

(4) $$BAI=\frac{Hip\;circumference(cm)}{{Height\left(m\right)}^{3/2}}-18$$

The indices used to evaluate insulin resistance are summarized in Table 1.

**Table 1:** Surrogate indices for measuring insulin resistance.

Index	HOMA-IR	QUICKI	Matsuda Index	TyG Index	TG/HDLc Ratio
Fasting glucose	X	X	-	X	
Fasting insulin	X		X		
Fasting triglycerides	-	-	-	X	X
Glucose tolerance test	-	-	X	-	
HDLc	-	-	-	-	X
Calculation formula	[glucose] (mmol/L) × [insulin] (µU/mL)/22.5 [94]	1/[log (lµU/mL) + log (log (G(mg/dL)))] [95]	10,000/√[(fasting G × fasting I) (mean G × mean I)] [65]	[ln (fasting triglycerides) (mg/dL) × fasting glucose (mg/dL)/2] [69]	TG/HDLc level
Insulin resistance	NV < 2.0–2.5	0.382 ± 0.007 for non-obese, 0.331 ± 0.01 for obese, and 0.304 ± 0.007 for diabetic individuals	<4.3 predicts IR	NV < 4 >9 [72,96]	Ideal: less than 2 Moderate risk: between 2 and 4 High risk: 4 or higher
Cost	Medium	Medium	High	Low	Low

HOMA—homeostasis model assessment; QUICKI—quantitative insulin sensitivity check index; TyG index—triglyceride and glucose index; TG/HDLc ratio—triglyceride to HDL-cholesterol ratio; NV—normal value; G—glucose.

The interrelationship between insulin resistance and atherosclerosis in humans is a critical area of study due to its significant implications for cardiovascular health [97]. Insulin resistance, a defining feature of type 2 diabetes and metabolic syndrome, is characterized by the body’s reduced ability to utilize insulin effectively, resulting in hyperinsulinemia and a series of metabolic disturbances. Conversely, atherosclerosis is a chronic inflammatory condition of the arterial walls, driven by the accumulation of lipids, immune cells, and fibrous tissue, leading to plaque formation and arterial stiffening. The interplay between these two conditions is intricate, involving multiple biochemical pathways and genetic factors that contribute to the progression of the disease [98].

## 5. Biochemical Pathways Linking Insulin Resistance to Atherosclerosis

Insulin resistance and atherosclerosis are two closely intertwined conditions that contribute significantly to cardiovascular morbidity and mortality. The relationship between these two conditions is complex and bidirectional, with insulin resistance exacerbating atherosclerosis and vice versa [99]. The molecular mechanisms underlying the insulin resistance-and-atherosclerosis relationship involve complex interactions between metabolic, inflammatory, and vascular pathways [100,101,102,103]. Insulin resistance creates a pro-atherogenic environment by altering lipid metabolism, promoting inflammation, and impairing vascular function. Understanding these mechanisms provides a foundation for developing targeted therapies to mitigate the burden of cardiovascular disease in insulin-resistant states.

### 5.1. Endothelial Dysfunction

#### 5.1.1. NO Pathway

Insulin resistance is intricately linked to endothelial dysfunction, a pivotal early event in the pathogenesis of atherosclerosis. The endothelium, a monolayer of cells lining the vasculature, plays a crucial role in maintaining vascular homeostasis by regulating vascular tone and inhibiting leukocyte adhesion and platelet aggregation. Insulin resistance compromises endothelial function by diminishing the bioavailability of nitric oxide, a potent vasodilator, while simultaneously augmenting the production of reactive oxygen species [104,105]. Insulin stimulates typically endothelial nitric oxide synthase (eNOS) activity via the phosphatidylinositol 3-kinase (PI3K)/Akt pathway, promoting NO production and vasodilation. Insulin resistance disrupts this pathway, leading to endothelial dysfunction and increased vascular tone [106,107].

This imbalance fosters oxidative stress, which subsequently activates pro-inflammatory pathways, including the nuclear factor-kappa B (NF-κB) pathway, resulting in the upregulation of adhesion molecules such as intercellular adhesion molecule-1 (ICAM-1) and vascular cell adhesion molecule-1 (VCAM-1) [108].

Nitric oxide (NO) is produced by endothelial cells and plays a key role in vasodilation, facilitating insulin delivery to skeletal muscles and other insulin-sensitive tissues. Reduced NO bioavailability impairs vasodilation, decreases insulin-mediated glucose uptake, and contributes to insulin resistance. Studies in healthy humans have demonstrated a positive correlation between endothelial NO production and insulin sensitivity, suggesting that NO deficiency directly impairs insulin action in the vasculature [109,110]. NO promotes insulin signaling by enhancing endothelial cell insulin uptake and trans-endothelial transport, partly through protein S-nitrosylation and the inhibition of protein tyrosine phosphatase 1B (PTP1B), which negatively regulates insulin signaling pathways. NO donors can restore insulin uptake and signaling impaired by inflammatory cytokines or high-fat diets, illustrating the protective role of NO against insulin resistance at the cellular level [111]. Insulin resistance, especially in type 2 diabetes, is associated with decreased nitric oxide synthase activity. This impairment contributes to vascular insulin resistance and metabolic dysfunction [112]. Some evidence suggests that reduced NO bioavailability in obesity and diabetes negatively affects insulin secretion, whereas other studies indicate that NO can inhibit glucose-stimulated insulin secretion. Restoring NO levels has shown beneficial metabolic effects in type 2 diabetes [113]. Nitric oxide deficiency contributes to insulin resistance primarily by impairing endothelial function, reducing insulin delivery to tissues, and disrupting insulin signaling pathways. This deficiency is common in obesity, type 2 diabetes, and related metabolic disorders. Strategies that increase NO bioavailability have potential therapeutic benefits for improving insulin sensitivity and preventing vascular complications associated with insulin resistance. This relationship highlights NO as a critical mediator linking vascular health and metabolic control in insulin-resistant states.

#### 5.1.2. Activity of Mitogen-Activated Protein Kinase (MAPK) Pathway

While PI3K pathways are blunted, insulin’s mitogen-activated protein kinase (MAPK)-dependent pathways remain intact, increasing endothelin-1 (ET-1) secretion, a potent vasoconstrictor. Compensatory hyperinsulinemia in insulin-resistant states further activates MAPK pathways, amplifying ET-1 release and upregulating adhesion molecules (e.g., VCAM-1, E-selectin), promoting monocyte adhesion and atherosclerosis [114,115].

MAPKs play a central role in endothelial dysfunction by mediating inflammatory and stress responses that disrupt endothelial cell function and vascular integrity [116]. MAPKs, including ERK1/2, JNK, and p38, regulate endothelial activation in response to pro-inflammatory stimuli. While ERK1/2 generally supports growth and cytoprotection, JNK and p38 are more involved in inflammatory and stress signaling. The balance and crosstalk among MAPKs influence the inflammatory status of endothelial cells, contributing to leukocyte migration and atherosclerosis progression [117]. The activation of p38 MAPK leads to the phosphorylation of microtubule-associated protein 4 (MAP4), which causes microtubule disassembly and endothelial barrier dysfunction [118]. The chronic activation of endothelial MAPK pathways results in increased vascular permeability, endothelial network disruption, and heightened inflammatory mediator production (e.g., sICAM, VCAM, IL-1β). This leads to vascular leakiness and inflammation-associated endothelial dysfunction, which can impair hematopoietic stem cell niches and promote myeloid-biased differentiation under stress conditions [119]. Altered MAPK signaling is implicated in the worsening of endothelial dysfunction in diseases such as insulin resistance, where vascular MAPK dysregulation exacerbates endothelial deterioration [120]. Their interaction with other inflammatory pathways like NF-κB further exacerbates endothelial injury, contributing to vascular diseases and impaired tissue homeostasis. This understanding positions MAPK pathways as potential therapeutic targets to restore endothelial function and vascular integrity in inflammatory and chronic vascular conditions [116,119,120,121].

### 5.2. Insulin Resistance and Inflammation

Insulin resistance and inflammation are closely interconnected, often creating a vicious cycle that exacerbates metabolic dysfunction and increases the risk of diseases like type 2 diabetes and cardiovascular conditions. Chronic inflammation, especially obesity-induced inflammation, is a key driver of insulin resistance. Inflammatory cytokines such as tumor necrosis factor-alpha (TNF-α) and interleukin-6 (IL-6), produced by adipose tissue and immune cells like macrophages, interfere with insulin signaling pathways in tissues including fat, muscle, and liver. This inhibition leads to reduced insulin sensitivity and impaired glucose metabolism [122,123]. When insulin signaling is impaired, it can increase the production of pro-inflammatory cytokines, creating a feedback loop where inflammation and insulin resistance worsen each other [124].

Macrophages are central to the pathophysiology of atherosclerosis, and insulin resistance markedly alters their function. Insulin-resistant macrophages demonstrate increased uptake of modified lipids, heightened production of inflammatory cytokines, and impaired efferocytosis (the clearance of apoptotic cells) [125,126]. This leads to the accumulation of apoptotic debris, necrotic core formation, and plaque instability. Furthermore, insulin resistance activates the unfolded protein response in macrophages, inducing apoptosis and further plaque necrosis [127,128].

In both conditions (atherosclerosis and insulin resistance) there is a switch in macrophage populations from an anti-inflammatory type (M2) to a pro-inflammatory type (M1), a switch that causes a vicious circle [129]. M1 macrophages ingest lipoproteins, which leads to the formation of foam cells. These cells are a key component of atherosclerotic plaques, which are deposits that form in the walls of arteries. These macrophages secrete pro-inflammatory cytokines like interleukin-6 (IL-6) and tumor necrosis factor alpha (TNFα), thereby amplifying the inflammatory response. In the context of insulin resistance, M1 macrophages increase tissue-based inflammation through the secretion of inflammatory markers. This inflammation can interfere with insulin signaling, worsening insulin resistance and contributing to the development of type 2 diabetes [130].

Insulin resistance associated with fibrous cap thinning was reported in various clinical studies, in which the necrotic core volume assessed both by coronary CT angiography and optical coherence tomography was positively correlated with HOMA-IR [131,132].

The mechanism of insulin resistance-mediated thin-cap fibroatheromata (TCFA) production involves the impairment of the PI3K/Akt pathway in macrophages, reducing cell survival and increasing endoplasmic reticulum (ER) stress. PI3K/ Akt pathway suppression leads to heightened vulnerability to ER stress-induced apoptosis. The accumulation of unfolded proteins (unfolded protein response) induced by free cholesterol loading or oxidized LDL triggers the CHOP (GADD153) pathway, augmenting macrophage death [133,134]. Insulin-resistant macrophages upregulate the scavenger receptor A, leading to more cholesterol uptake, generating a positive feedback loop that augments macrophage apoptosis. An impaired MEK-ERK pathway triggers defective SERCA signaling. Increased intracellular calcium concentration triggers the formation of the mitochondrial permeability transition pore (MPTP), allowing cytochrome c to be released into the cytoplasm which, in turn, triggers caspase-mediated apoptosis. The accumulation of dead cells increases the necrotic core volume, exacerbating inflammation and plaque instability 5. Furthermore, foam cell apoptosis releases free cholesterol in the plaque microenvironment, leading to cholesterol crystal accumulation [135]. Cholesterol crystals are phagocytosed by macrophages and disrupt phagolysosomal integrity, leading to cytoplasmic cathepsin leakage. Cathepsin leakage augments the NLRP3 inflammasome assembly, leading to increased IL-1β and IL-18 release [136]. Both IL-1β and IL-18 activate monocytes and T cells to secrete additional inflammatory mediators, such as IL-6 and interferon-γ, perpetuating local vascular inflammation [137,138].

Adaptive immunity also influences plaque stability by the T cell and B cell receptor-mediated recognition of epitopes from oxidized LDL particles and heat shock proteins. IL-18 promotes Th1 cell differentiation with further interferon-γ production and macrophage activation [139]. Furthermore, IL-1β production activates Th17 lymphocytes, enhancing the recruitment and activation of inflammatory cells, including neutrophils and monocytes. This, in turn, augments vascular inflammation and accelerates atherosclerotic plaque development. Regulatory T cells (Tregs), CD4+, CD25+, and Foxp3+ play a crucial role in suppressing inflammation and atherosclerosis by inhibiting pro-inflammatory immune cells and releasing anti-inflammatory cytokines like TGF-β and IL-10. They help maintain plaque stability by promoting collagen production and reducing plaque inflammation, while their depletion accelerates lesion formation. The balance between Tregs and pro-inflammatory T cells, especially Th1 cells, determines atherosclerosis progression, with Tregs counteracting lesion growth and promoting plaque regression through inflammation resolution and tissue repair. Additionally, Tregs encourage macrophage polarization toward the anti-inflammatory M2 phenotype, enhancing the clearance of dead cells and further supporting plaque stabilization and regression. Supporting these mechanisms, a study by Liuzzo et al. showed that patients with acute coronary syndromes expressed a higher proportion of Th1 lymphocytes, while the Treg population was poorly represented [140]. The explanation for the altered Th1/Treg ratio in atherosclerotic conditions might be attributed to the downregulation of Foxp3 and CD25, leading to an impaired anti-inflammatory response. Meanwhile, Th1 cells expand and secrete IFN-γ and TNF-α, which drive macrophage activation, endothelial dysfunction, and plaque instability. This shift toward a dominant pro-inflammatory Th1 response and diminished anti-inflammatory Treg activity exacerbates vascular inflammation, lesion progression, and plaque vulnerability [141,142,143].

**Figure 2:**
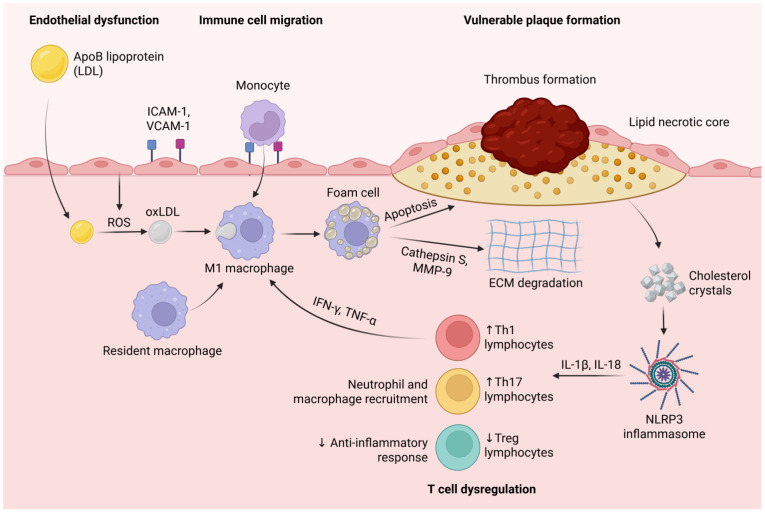
Endothelial dysfunction promotes the infiltration of ApoB-containing lipoproteins (LDLs) into the arterial wall, where they undergo oxidative modification (oxLDL) via reactive oxygen species (ROS). OxLDL triggers the recruitment of monocytes through the upregulation of adhesion molecules (ICAM-1, VCAM-1). Monocytes differentiate into resident and M1 macrophages, which engulf oxidized LDL (oxLDL) to form foam cells. Pro-inflammatory cytokines (IFN-γ, TNF-α) secreted by Th1 lymphocytes further activate M1 macrophages, amplifying inflammation. Foam cell apoptosis and the secretion of proteases such as cathepsin S and MMP-9 contribute to extracellular matrix (ECM) degradation, the thinning of the fibrous cap, and the expansion of the lipid necrotic core. Cholesterol crystals released from dying foam cells activate the NLRP3 inflammasome, leading to increased secretion of IL-1β and IL-18, which further recruit inflammatory cells and perpetuate vascular inflammation. The dysregulation of T cell subsets-characterized by increased Th1 and Th17 lymphocytes and decreased regulatory T cells (Tregs) exacerbates inflammation, disrupts anti-inflammatory responses, and promotes plaque instability and thrombus formation. Created in BioRender. Tirziu, A. (2025) https://BioRender.com/lcalh3v (accessed on 20 May 2025).

Additionally, insulin resistance augments reactive oxygen species production, leading to an imbalance between matrix metalloproteinases (particularly MMP-9) and their inhibitors (TIMPs). This imbalance, despite facilitating smooth muscle cell migration and plaque expansion, makes atherosclerotic plaque more prone to rupture, leading to acute cardiovascular events [144,145] (Figure 2).

### 5.3. Dyslipidemia and Lipid Metabolism

Insulin resistance is frequently associated with dyslipidemia, characterized by elevated triglycerides, LDL-C, and small, dense LDL particles. These lipid abnormalities facilitate the infiltration of LDL into the arterial wall, where it undergoes oxidation (oxLDL) and is subsequently internalized by macrophages via scavenger receptors, culminating in foam cell formation and the initiation of atherosclerotic plaques [146,147]. Insulin resistance impairs the regulation of lipid metabolism, leading to increased free fatty acid flux from adipose tissue to the liver. This results in the overproduction of very-low-density lipoprotein (VLDL) and the accumulation of triglyceride-rich lipoproteins in the bloodstream. Triglyceride-rich lipoproteins are not just markers but are likely causal risk factors for inflammation, atherosclerotic cardiovascular disease, and even all-cause mortality. Extensive population studies have shown that higher levels of triglycerides and remnant cholesterol are linked with significantly increased risks of myocardial infarction, ischemic heart disease, ischemic stroke, and overall mortality [148,149].

### 5.4. Hepatic Insulin Resistance and Inflammation

Hepatic insulin resistance is a critical contributor to systemic inflammation and atherosclerosis. In response to insulin resistance, the liver synthesizes pro-inflammatory cytokines and acute-phase reactants such as CRP. These factors exacerbate endothelial dysfunction, enhance the expression of adhesion molecules, and increase the uptake of oxidized LDL by macrophages, all of which contribute to the formation and progression of atherosclerotic plaques [150,151].

### 5.5. The Role of Estrogen Deficiency

Before menopause, women typically experience a lower incidence of cardiovascular disease (CVD) compared to men; however, this gap narrows significantly after menopause. This dysfunction tends to worsen following menopause. Estrogen plays a vital role in maintaining endothelial health by promoting the production of nitric oxide (NO), a key vasodilator that helps relax blood vessels and sustain vascular tone. Additionally, it reduces levels of endothelin-1, a potent vasoconstrictor and pro-inflammatory peptide produced by the endothelium [152].

The decline in estrogen levels during menopause reduces the availability of NO and increases oxidative stress, contributing to endothelial dysfunction [153]. Experimental and clinical studies show that estrogen deficiency increases vascular free radical production and enhances vasoconstriction mediated by angiotensin II, further contributing to endothelial dysfunction [154,155]. Clinical evidence indicates that estrogen replacement therapy can improve endothelial function in postmenopausal women by restoring NO-mediated vasodilation and reducing oxidative stress. However, the efficacy of such treatment may depend on timing and receptor status [153,156].

### 5.6. Role of Vascular Smooth Muscle Cells (VSMCs)

VSMCs play a significant role in the relationship between insulin resistance and vascular disease, particularly atherosclerosis and remodeling. Under normal conditions, insulin maintains VSMC quiescence (a non-proliferative state) and modestly stimulates migration. Insulin promotes VSMC differentiation by increasing alpha-smooth muscle actin (alpha-SMA) expression, a marker of the contractile phenotype. However, insulin can also enhance VSMC migration through the MAP kinase pathway [157]. Insulin resistance in VSMCs disrupts normal insulin signaling pathways, particularly those involving insulin receptor substrate-1 (IRS-1) and downstream PI3K-Akt signaling. Angiotensin II (ANG II) induces the phosphorylation and degradation of IRS-1 in VSMCs, impairing insulin signaling and reducing glucose uptake [158]. The loss of normal insulin receptor signaling in VSMCs leads to increased proliferation and migration, key processes in atherosclerosis progression. VSMCs lacking insulin receptors show resistance to mTOR inhibition, suppressing typical proliferation and migration, thus exacerbating vascular disease [159]. Insulin resistance in VSMCs involves selective impairment of the PI3K-Akt pathway (responsible for metabolic actions like glucose uptake).

In contrast, the MAPK pathway (linked to mitogenic and inflammatory responses) remains active or is upregulated. This imbalance promotes VSMC proliferation, migration, and inflammation, contributing to vascular pathology. Therapeutic agents that improve insulin sensitivity, such as PPAR-γ agonists (e.g., pioglitazone), can help restore insulin signaling balance in VSMCs, reducing pathological proliferation and migration and potentially slowing atherosclerosis progression [160].

### 5.7. The Role of Maladaptive Responses to the Disruption of Cellular Homeostasis

Maladaptive responses to cellular stress are central to the progression from temporary dysfunction to chronic disease. They represent a breakdown in the cell’s capacity to restore homeostasis, often resulting in tissue injury, inflammation, and organ dysfunction [161].

Maladaptive stress responses in insulin resistance involve the following: 1. Cellular Stresses (factors such as nutrient overload, physical inactivity, hypoxia, and environmental toxins induce cellular stresses, including oxidative, nitrosative, carbonyl, genotoxic, and endoplasmic reticulum stress). 2. Stress Response Pathways. (Cells initiate adaptive responses like the unfolded protein response, DNA damage response, and autophagy to restore homeostasis. However, chronic or excessive activation leads to maladaptive outcomes-persistent inflammation, apoptosis, and impaired insulin signaling.) 3. Beta Cell Dysfunction (in pancreatic beta cells, maladaptive responses to chronic stress result in impaired insulin secretion, dedifferentiation, and apoptosis, contributing to the progression from compensation to decompensation in insulin resistance and type 2 diabetes). 4. Trained Immunity (insulin resistance alters immune cell metabolism, particularly in macrophages, promoting a state of “trained immunity” that sustains maladaptive inflammatory responses and further disrupts metabolic homeostasis) [162].

Maladaptive stress responses in atherosclerosis involve the following: 1. Endoplasmic Reticulum Stress. (Chronic ER stress in vascular cells triggers the UPR. If unresolved, this leads to apoptosis, inflammation, mitochondrial dysfunction, and oxidative stress—all key drivers of atherosclerotic plaque formation and instability). 2. Mechanical Forces and Vascular Homeostasis. (Normally, endothelial and vascular smooth muscle cells adapt to mechanical forces like shear stress to maintain vascular health. Chronic disturbed flow or other risk factors can make these adaptive responses maladaptive, resulting in the sustained production of reactive oxygen species (ROS), activating pro-inflammatory pathways (MAPK, NF-κB) and promoting a pro-atherogenic state). 3. Unresolved Inflammation (atherosclerosis is now understood as a chronic inflammatory disease, in which maladaptive cellular responses fail to resolve inflammation, perpetuating vascular injury and plaque progression) [163].

Insulin resistance and atherosclerosis involve maladaptive cellular responses to metabolic and environmental stressors, particularly chronic inflammation, oxidative stress, and impaired autophagy [162]. While insulin resistance and atherosclerosis can develop independently, maladaptive stress responses in one can exacerbate the other. For example, insulin resistance promotes vascular inflammation and endothelial dysfunction, accelerating atherosclerosis [164]. Maladaptive cellular responses to disrupted homeostasis are central to developing and progressing insulin resistance and atherosclerosis. These maladaptive processes, originally evolved as adaptive mechanisms to restore balance, become detrimental when chronically activated, leading to persistent inflammation, cellular dysfunction, and ultimately, disease [163,165].

## 6. Genetic Factors in Insulin Resistance and Atherosclerosis

### 6.1. Genetic Predisposition to Metabolic Syndrome

Family history and genetic predisposition significantly contribute to the development of insulin resistance and atherosclerosis. Numerous genetic markers associated with insulin resistance and cardiovascular disease have been identified, including variants in the *IRS1*, *CHI3L1*, and *CD36* genes [166,167]. These genetic factors influence lipid metabolism, inflammatory responses, and insulin signaling pathways, thereby creating a predisposition to both conditions.

### 6.2. Haplotypes and Protective Genetic Variants

Specific haplotypes upstream of the insulin receptor substrate 1 (*IRS1*) gene are linked to insulin resistance, type 2 diabetes, and atherosclerosis. For instance, one haplotype has been found to confer protection against insulin resistance, adverse lipid profiles, and asymptomatic atherosclerosis, indicating a genetic connection between these conditions [168]. These findings underscore the potential of genetic screening and personalized therapies in managing insulin resistance and atherosclerosis.

### 6.3. Epigenetic and Environmental Interactions

Epigenetic modifications, such as DNA methylation and histone acetylation, also contribute to the development of insulin resistance and atherosclerosis. These changes are influenced by environmental factors, including diet and lifestyle, and may regulate the expression of genes involved in insulin signaling and lipid metabolism. Understanding these interactions is essential for developing targeted interventions to mitigate the risk of atherosclerosis in insulin-resistant individuals [169].

## 7. Is There a Link Between Insulin Resistance and ATS?

Numerous studies have identified a correlation between insulin resistance and cardiovascular risk [170,171]. Insulin resistance results in elevated insulin levels and hyperglycemia, even prior to the onset of diabetes mellitus [172]. A recent meta-analysis demonstrated that increased insulin levels and hyperglycemia, even in individuals without diabetes, are associated with heightened cardiovascular risk [173]. Insulin resistance may contribute to atherosclerotic disease through mechanisms such as dyslipidemia, endothelial dysfunction, hypertension, and inflammation, although these mechanisms are not yet fully elucidated [133,171,174].

In the Multi-Ethnic Study of Atherosclerosis (MESA), insulin resistance, as measured by HOMA-IR, was correlated with subclinical ATS, as indicated by IMT or the coronary calcium score, but only in the presence of metabolic syndrome [175]. Conversely, in a cohort of 3741 patients with HbA1c < 6% and no known cardiovascular disease, insulin resistance measured by HOMA-IR > 3 was correlated with subclinical ATS, as evidenced by carotid IMT or the coronary calcium score [176]. A recent study identified a correlation between insulin resistance (defined as HOMA-IR > 2.5) and subclinical ATS (measured by carotid IMT or ankle–brachial index), independent of other risk factors [177].

Another recent study evaluated the correlation between insulin resistance (characterized by the HOMA-IR index, TyG index, and triglyceride levels) and subclinical ATS (detected by the calcium score and IMT in the carotid arteries), as well as arterial stiffness (measured by brachial–ankle pulse wave velocity (baPWV)). Upon reviewing several studies, it was concluded that the TyG index positively correlated with the calcium score, and both the TyG index and triglyceride levels correlated with baPWV. However, carotid artery IMT indices did not exhibit a positive correlation with any measure of insulin resistance. It was concluded that, among the measurements used to detect insulin resistance, the TyG index correlated most effectively with subclinical ATS, and its introduction into clinical practice was proposed [178]. The integration of the TyG index into current practice could enhance cardiovascular risk assessment, although this requires confirmation in future studies, both as a marker and therapeutic target [89,178].

## 8. Therapeutic Strategies for Insulin Resistance

Insulin resistance generates a high burden of cardiovascular diseases, as a 10-year history of type 2 diabetes is associated with a 20% risk of experiencing a myocardial infarction and a 40% likelihood of MI recurrence [179]. Currently, there is no specific pharmacological treatment for insulin resistance; however, it can be modulated through lifestyle modifications, increased physical activity, or certain medications [180,181,182]. Various drug classes have been identified that enhance insulin sensitivity in patients with prediabetes or diabetes mellitus, including biguanides, thiazolidinediones, GLP-1 receptor agonists, and SGLT2 inhibitors. Metformin is one such drug with evidence supporting its ability to improve insulin sensitivity through multiple mechanisms [183]. It has been shown to enhance insulin sensitivity in individuals with prediabetes, type 2 diabetes mellitus, obesity, polycystic ovary syndrome, and non-alcoholic hepatic steatosis by increasing peripheral glucose utilization, augmenting insulin receptor tyrosine kinase activity, promoting glycogen synthesis, and enhancing the recruitment and activity of GLUT4 [184]. Some studies have concluded that SGLT2 inhibitors, when administered to patients with type 2 diabetes mellitus, ameliorate insulin resistance (as measured by HOMA-IR) primarily by increasing glucose uptake in skeletal muscle and promoting lipolysis in adipose tissue. GLP-1 receptor agonists (liraglutide, semaglutide, and exenatide) have been found to inhibit macrophage inflammatory responses, thereby reducing insulin resistance [185]. Additionally, these agents facilitate weight loss and are recommended for obesity treatment, with improvements in insulin sensitivity observed prior to significant weight reduction [186,187].

Tirzepatide, a dual GIP and GLP-1 receptor agonist approved by the FDA in 2022, has shown significant improvements in glycemic control and weight reduction without increasing hypoglycemia risk, further improving insulin resistance in T2D patients [188].

Dipeptidyl peptidase 4 inhibitors (DPP-4i) (sitagliptin, vildagliptin, and linagliptin) have been shown to enhance insulin resistance in patients with type 2 diabetes mellitus by mitigating macrophage-induced inflammation without affecting insulin secretion [189,190,191]. Pioglitazone improves insulin sensitivity by reducing muscle lipids and reorganizing lipids in adipose tissue [192], thereby decreasing the risk of major cardiovascular events (myocardial infarction and nonfatal stroke) in patients with prediabetes and diabetes, although it is associated with an increased risk of heart failure, bone fractures, and weight gain [193]. A pertinent question is whether insulin administration is advantageous for patients with type 2 diabetes mellitus and insulin resistance. This question was addressed in a study where, in patients with high insulin resistance (measured by HOMA-IR, with the cut-off point for high insulin resistance being the median = 4.55; interquartile range = [2.39–7.91]), exogenous insulin administration was associated with an elevated risk of mortality or major cardiovascular events, as well as a deterioration in renal function [194].

A groundbreaking new treatment called ReCET (Re-Cellularization via Electroporation Therapy) was presented in 2024. This procedure, performed under deep sedation, aims to improve the body’s sensitivity to its own insulin, addressing the root cause of insulin resistance. When combined with semaglutide, a GLP-1 receptor agonist, this approach eliminated the need for insulin therapy in 86% of patients in a small first-in-human study. Larger trials are underway to validate these promising results [195]. PATAS is an emerging medication in development that targets insulin resistance by correcting abnormalities in fat cells. It works by separating two proteins that block insulin’s action, thereby allowing fat cells to absorb glucose without needing insulin. This treatment could prevent T2D and other insulin resistance-related conditions like fatty liver and metabolic syndrome. Human trials are expected to begin in 2025, and it may be administered via injection or patch [196].

## 9. Conclusions

The intricate relationship between insulin resistance and atherosclerosis is increasingly recognized as a pivotal factor in the development and progression of cardiovascular disease. Insulin resistance independently predicts the progression of atherosclerotic plaques, even in individuals without diabetes, and acts through both traditional cardiovascular risk factors and direct vascular effects. The early detection of both subclinical ATS and IR is essential, as these conditions often precede overt clinical disease by years, providing a critical window for intervention.

The diagnosis of IR in clinical practice relies on surrogate indices such as HOMA-IR, the QUICKI, and the TyG index, which are helpful for screening and risk assessment. The early detection of ATS can be achieved through a combination of non-invasive imaging techniques (such as coronary artery calcium scoring and carotid ultrasound) and comprehensive risk profiling, which should include the identification of insulin resistance. However, the heterogeneity in diagnostic thresholds and the influence of population-specific factors highlight the need for standardized approaches and further research. Understanding and targeting the IR–ATS axis is essential for the effective prevention and management of cardiovascular disease, as interventions aimed at ameliorating insulin resistance may have a significant impact on reducing cardiovascular risk. Genetic factors and changes that affect gene expression play a role in both insulin resistance and atherosclerosis, suggesting that personalized medicine may become increasingly important in managing cardiovascular risk. This integrated approach would help clinicians identify high-risk individuals earlier and personalize treatment strategies accordingly. Future research should focus on refining diagnostic criteria, elucidating underlying mechanisms, and evaluating the impact of early, routine screening for IR in diverse populations.

The evidence supports a direct and clinically significant link between insulin resistance and atherosclerosis and highlights the importance of early detection and intervention in individuals at risk. Integrating the early detection and management of insulin resistance into cardiovascular prevention frameworks is crucial to mitigating the morbidity and mortality associated with atherosclerosis. Further research is required to investigate the interplay between metabolic and vascular health, which will be vital for developing more effective, individualized strategies for cardiovascular risk reduction.

## Abbreviations

The following abbreviations are used in this manuscript:

Abbreviation	Full Term
ATS	Atherosclerosis
GLUT	Glucose Transporter (e.g., GLUT-2, GLUT-3, GLUT-4)
LDL	Low-Density Lipoprotein
LDLc	Low-Density Lipoprotein Cholesterol
HDL	High-Density Lipoprotein
HDLc	High-Density Lipoprotein Cholesterol
TG	Triglyceride
TyG	Triglyceride Glucose Index
CAD	Coronary Artery Disease
CT	Computed Tomography
JACC	Journal of the American College of Cardiology
Lp(a)	Lipoprotein(a)
MMP-9	Matrix Metalloproteinase-9
IMT	Intima-Media Thickness
CPs	Carotid Plaque Score
cIMT	Combined Intima-Media Thickness
baPWV	Brachial–Ankle Pulse Wave Velocity
HOMA-IR	Homeostatic Model Assessment for Insulin Resistance
QUICKI	Quantitative Insulin Sensitivity Check Index
G	Glucose (in the Context of the Matsuda Index Formula)
I	Insulin (in the Context of the Matsuda Index Formula)
ln	Natural Logarithm
log	Base 10 logarithm
